# A strong summer phytoplankton bloom southeast of Vietnam in 2007, a transitional year from El Niño to La Niña

**DOI:** 10.1371/journal.pone.0189926

**Published:** 2018-01-17

**Authors:** Hui Zhao, Jian Zhao, Xingli Sun, Fajin Chen, Guoqi Han

**Affiliations:** 1 Guangdong Ocean University, Zhanjiang, China; 2 Northwest Atlantic Fisheries Centre, Fisheries and Oceans Canada, St. John’s, NL, Canada; CAS, CHINA

## Abstract

Summer upwelling occurs frequently off the southeast Vietnam coast in the western South China Sea (SCS), where summer phytoplankton blooms generally appear during June-August. In this study, we investigate inter-annual variation of Ekman pumping and offshore transport, and its modulation on summer blooms southeast of Vietnam. The results indicate that there are low intensities of summer blooms in El Niño years, under higher sea surface temperatures (SST) and weaker winds. However, a different pattern of monthly chlorophyll a (Chl-a) blooms occurred in summer of 2007, a transitional stage from El Niño to La Niña, with weak (strong) wind and high (low) SST before (after) early July. There is a weak phytoplankton bloom before July 2007 and a strong phytoplankton bloom after July 2007. The abrupt change in the wind intensity may enhance the upwelling associated with Ekman pumping and offshore Ekman transport, bringing more high-nutrient water into the upper layer from the subsurface, and thus leading to an evident Chl-a bloom in the region.

## Introduction

Upwelling refers to the upward motion of the water body that brings subsurface water to the sea surface, with the surface water taken away from upwelling regions through horizontal advection [1]. Upwelling can promote mixing of deep water with surface water, which leads to changes in physical and chemical properties (e.g. temperature) of seawater in the area [2]. The wind pattern in the South China Sea (SCS) is mainly controlled by the East Asian monsoon, with southwesterly and northeasterly monsoonal winds in summer and winter, respectively. During the southwest monsoon, summer upwelling appears generally in the northern shelf and the western SCS through offshore Ekman transport induced by the wind stress component parallel to the coastlines and through Ekman pumping caused by wind stress curls [3,4].

A statistical correlation exists between the wind stress and the sea surface temperature (SST) on various spatial and temporal scales [3,5]. The magnitude of the wind has a positive effect on upwelling [6]. In the Pearl River plume, wind direction exerted a more substantial effect on upwelling than wind speed [7]. Upwelling intensity can be estimated by observation of SST anomalies, since the upwelling brings low-temperature water from deep layer into the surface [8]. The upwelling intensity is also affected by the terrain. Because of the conservation of the potential vorticity, the terrain is positively correlated with the upwelling [9]. Upwelling variation can also be highly coupled with climate change [10]. Increasing greenhouse gases enhance the upwelling of the coast of northern California and other similar upwelling regions [11]. Moreover, upwelling events have quasi-instantaneous and cumulative effects on the intertidal water, leading to generally colder temperatures. Most of the world's largest fisheries are located in the upwelling regions [12]. The occurrence and disappearance of upwelling has also affected development of fishery production [13]. Therefore, upwelling can exert a significant influence on fisheries and marine ecosystems.

Phytoplankton is a primary producer in marine ecosystems, and plays a vital role in the marine food chain/web. It is also an important intermediary for the conversion of inorganic matter into organic matter. The chlorophyll-a concentration (Chl-a) can represent phytoplankton biomass and in turn the level of primary productivity in the seawater. Recent studies of the SCS indicate that high Chl-a concentrations were located in the coastal zones with strong upwelling [14–16]. Lin et al. [17] studied the relationship between Chl-a and SST, and found a negative correlation between Chl-a concentration and SST. Zhao et al. [18] analyzed the interannual variability of Chl-a concentration in the SCS and discussed possible impact of upwelling and offshore currents on Chl-a concentration changes. Chen et al. [19] studied the relationship between nutrients and Chl-a concentration in spring. Using remote sensing ocean color data, Tang et al. [20,21] analyzed the effect of upwelling on algal blooms northwest of Luzon Island and southeast of Vietnam, respectively. Finally, Zhao and Tang [4] discussed the relationship between Chl-a concentration and El Niño, wind direction, and Ekman pumping, based on a case study for an El Niño year.

**Fig 1:**
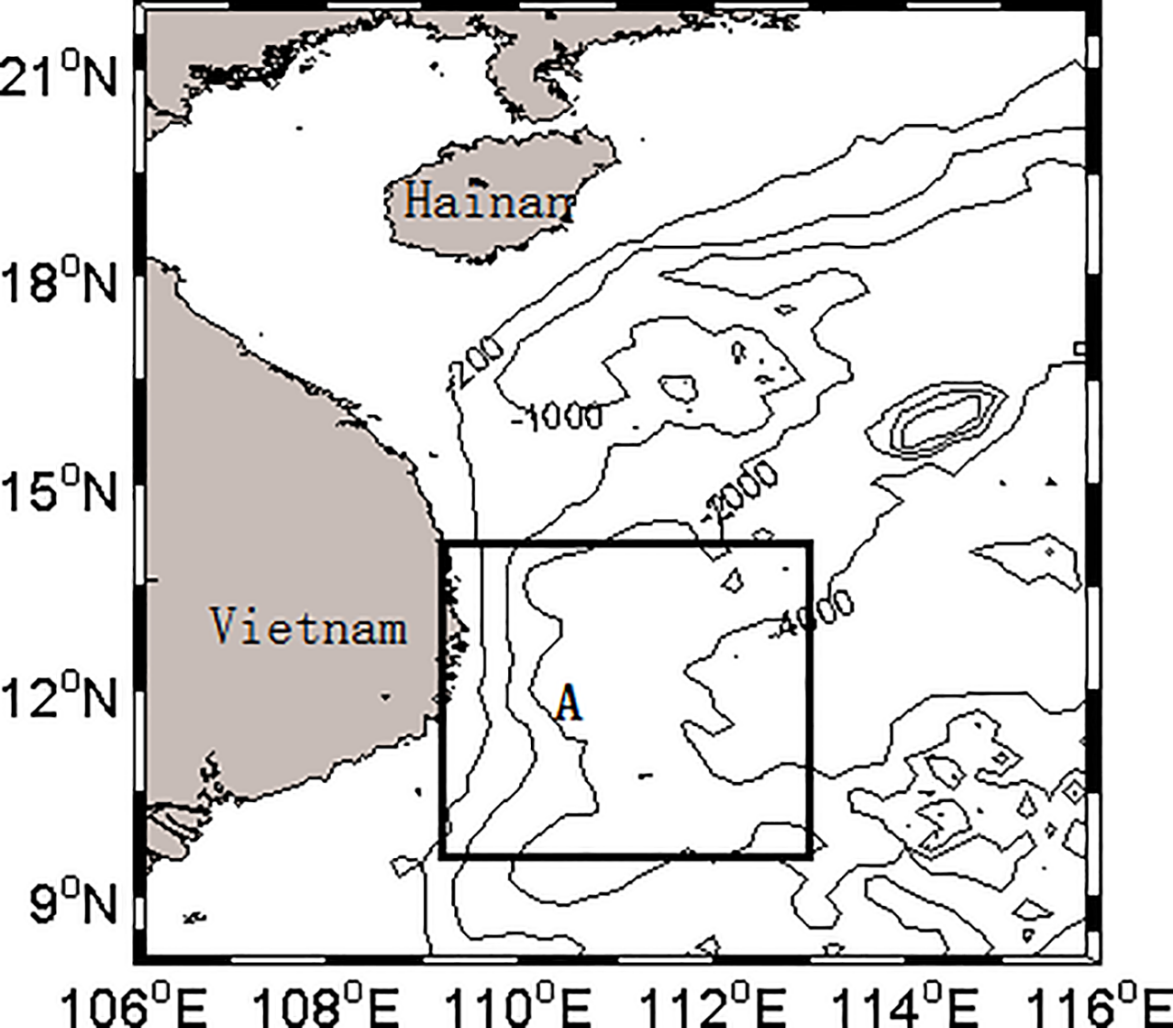
Location of the study area in the South China Sea. Box A is the upwelling area southeast of Vietnam.

The present study region is located in the western SCS (8°-22°N, 106°-116°E; Fig 1). In summer, the southwest monsoon can trigger upwelling in this area (box A in Fig 1) [22–23], which exerts an important influence on phytoplankton and marine ecosystems in this area. The upwelling southeast of Vietnam [24] could accelerate the transport of deep nutrient-rich water into the surface, which promoted the growth of phytoplankton, enhancing the primary productivity of the waters, and further enriched the diversity of the whole species. In this paper, we investigate the inter-annual changes of phytoplankton blooms and responses of Chl-a patterns to the summer upwelling In addition, we also examine other ocean conditions in this area, to discuss the different roles that the summer upwelling (and other ocean conditions) plays in increasing the concentration of phytoplankton and Chl-a.

## Data and methods

### Satellite data

#### Merged multi-sensor Chl-a data

Chl-a satellite fusion data products derived from the SeaWiFS, MODIS, MERIS and VIIRS products (available at http://hermes.acri.fr) are acquired, which are processed by the semi-analytical Garver Siegel Maritorena (GSM) algorithm [25]. The normalized reflectance values at the original sensor wavelengths are used in the GSM method. The monthly L3 products of Chl-a, with a spatial resolution of 4 km, are used for the period of Jan. 1998—Dec. 2014 in the study. The daily Chl-a data from June 1 to August 31, with a spatial resolution of 4 km, were also utilized for the same period, to investigate further variation of Chl-a in a specific year.

#### Sea surface wind (SSW) and sea surface temperature (SST)

SSW and SST data are derived from European Centre for Medium-Range Weather Forecasts with the spatial resolutions of 0.125° ×0.125°. The monthly products of SSW and SST are used for the period from Jan. 1998 to Dec. 2014. The daily products of SSW and SST are also used in the summers (i.e. June 1 to August 31) between 1998 and 2014.

#### Precipitation

Precipitation data are acquired from the Tropical Rainfall Measuring Mission (TRMM), which is provided at 0.25° by 0.25° spatial resolutions (http://data.remss.com/tmi/bmaps_v07.1/). The daily product of summer precipitation is also utilized for the years from 1998 to 2014.

#### Geostrophic velocities

Geostrophic velocity data with a spatial resolution of 0.25° from January 1998 to December 2014 were obtained from the Archiving, Validation and Interpretation of Satellite Oceanographic dataset (AVISO) (http://www.aviso.altimetry.fr).

### Methods

#### Method of estimating the upwelling velocity

In this study, we estimate the Ekman pumping velocity (EPV) and the coastal upwelling compensation for the offshore Ekman transport (ET) following Halpern [26], as shown in Eq (1) and Eq (2) below,

$$EPV=\frac{curl(\tau )}{\rho f}+\frac{\beta \tau_{x}}{\rho f^{2}}$$ (1)

$$E_{}T=\frac{\tau_{c}}{\rho_{}f}$$ (2)

where τ_c_ is the wind stress parallel to the coastline, τ_x_ is the wind stress perpendicular to the coastline, ρ is the sea water density, *f* is the Coriolis parameter, and β is the gradient of the Coriolis parameter *f*. We neglect the influence of the Coriolis force gradient β because it is a small value in the study region.

#### Multiple correlation analysis

A multiple correlation analysis is used to reveal the roles of SST, ET, and EPV in determining the magnitude of the phytoplankton increase in the region southeast of Vietnam. The correlation matrix between variables of *x_1_,x_2_,…,x_m_,y* is defined as [27, 28],

$$R=\left[\begin{array}{ccccc} r_{11} & r_{12} & \ldots & r_{1m} & r_{1y}\\ r_{21} & r_{22} & \ldots & r_{2m} & r_{2y}\\ \vdots & \vdots & \vdots & \vdots & \vdots \\ r_{m1} & r_{m2} & \cdots & r_{mm} & r_{my}\\ r_{y1} & r_{y2} & \cdots & r_{ym} & r_{yy} \end{array}\right]$$ (3)

The correlation coefficients R_y·1,2,…m_ and partial correlation coefficients R_yi·1,2,…m_ between *x_i_* and *y* are written as follows:

$$R_{y•1,2,\ldots m}=\sqrt{1-\frac{\left|R\right|}{R_{yy}}}$$ (4)

$$R_{yi•1,2,\cdots ,m}=-\frac{R_{yi}}{\sqrt{R_{yy}R_{ii}}}$$ (5)

where *y* is a dependent variable, representing Chl-a in this paper, and *x_1_,x_2_,…x_m_* are the independent variables, representing SST, ET, and EPV. |R| is the determinant of a matrix R; R_*yy*,_,R_*ii*_ and R_*yi*_ are the algebraic complements of *r_yy_*, *r_ii,_* and r_yi_ in |R|, respectively. Here, *r_ij_* is the simple correlation among *x_1_,x_2_,…x_m_, y*.

#### Empirical orthogonal function analysis

EOF methods can be used to decompose a time series of a physical field into independent spatial distribution modes and temporal modes. The spatial modes (*l*_*ik*_) characterize the geographical distribution of the field, and the time functions (*y*_*kj*_) describe temporal variability of the spatial modes. The temporal modes are also called the principal component:

$$\begin{array}{l} x_{ij}=\sum_{k=1}^{m}l_{ik}y_{kj}=l_{i1}y_{1j}+l_{i2}y_{2j}+\cdots +l_{im}y_{mj}\\ \left(i=1,2,\cdots m,j=1,2,\cdots ,n\right) \end{array}$$ (6)

By dividing the variance contribution rate, we can decompose the physical field into several principal modes with the highest variances,

X=LY (7)

$$L=\left[\begin{array}{cccc} l_{11} & l_{12} & \cdots & l_{1m}\\ l_{21} & l_{22} & \cdots & l_{2m}\\ \vdots & \vdots & \vdots & \vdots \\ l_{m1} & l_{m2} & \cdots & l_{mm} \end{array}\right]$$ (8)

$$Y=\left[\begin{array}{cccc} y_{11} & y_{12} & \cdots & y_{1n}\\ y_{21} & y_{22} & \cdots & y_{2n}\\ \vdots & \vdots & \vdots & \vdots \\ y_{m1} & y_{m2} & \cdots & y_{mn} \end{array}\right]$$ (9)

In our study, we only retain the first mode of the EOF results since 70% of the variance in both SST and Chl-a are first-mode.

## Results

### Distribution of summer Chl-a concentration in the western SCS

**Fig 2:**
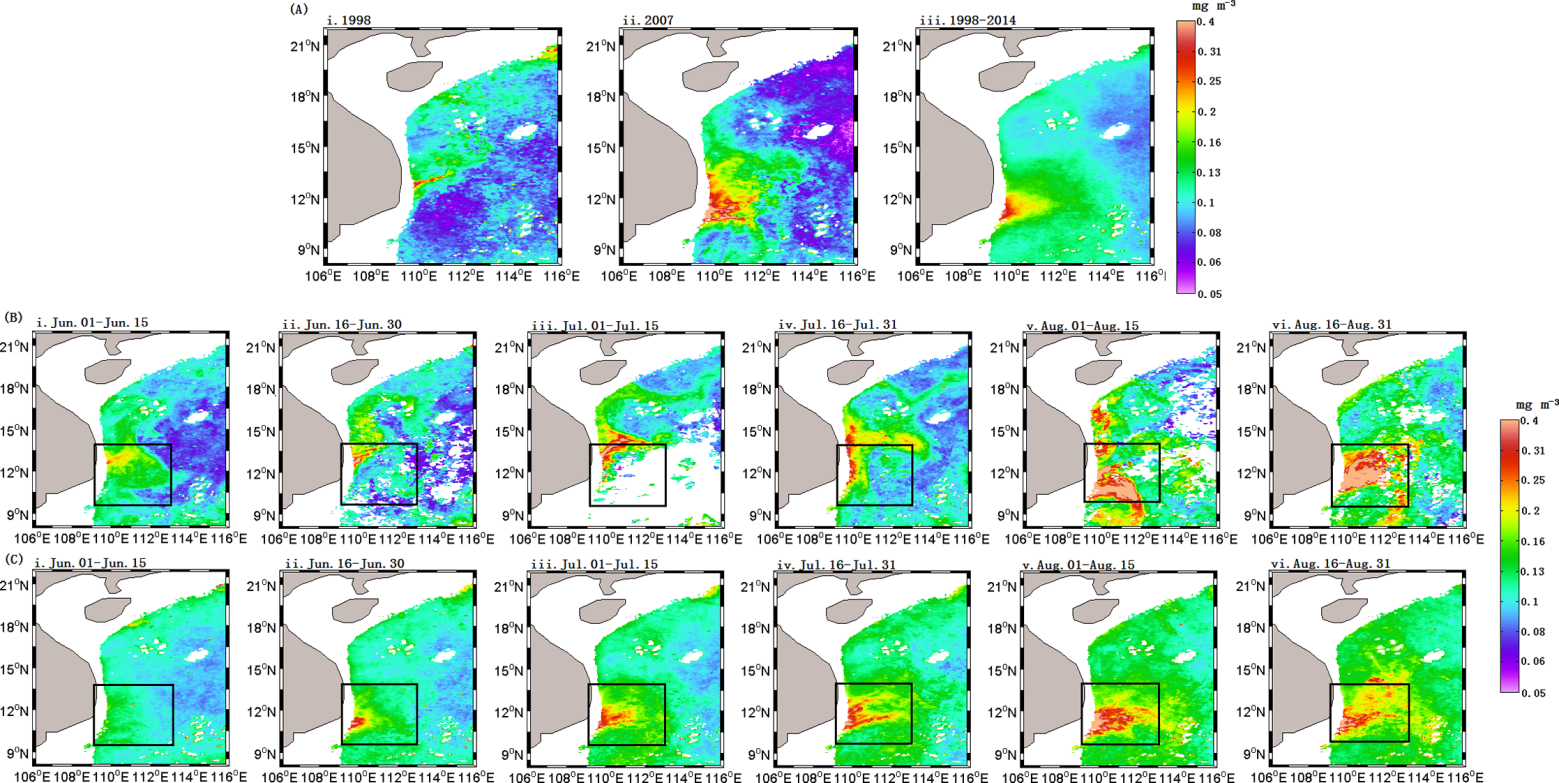
Spatial distribution of summer Chl-a concentration in the SCS. (A) Summer Chl-a (averaged for 1 June—31 August), for 1998 A(i), 2007 A(ii) and 1998–2014 A(iii); (B) and (C) Chl-a averaged for 15 days during summer of 2007 and climatology of 1998–2014, respectively.

**Fig 3:**
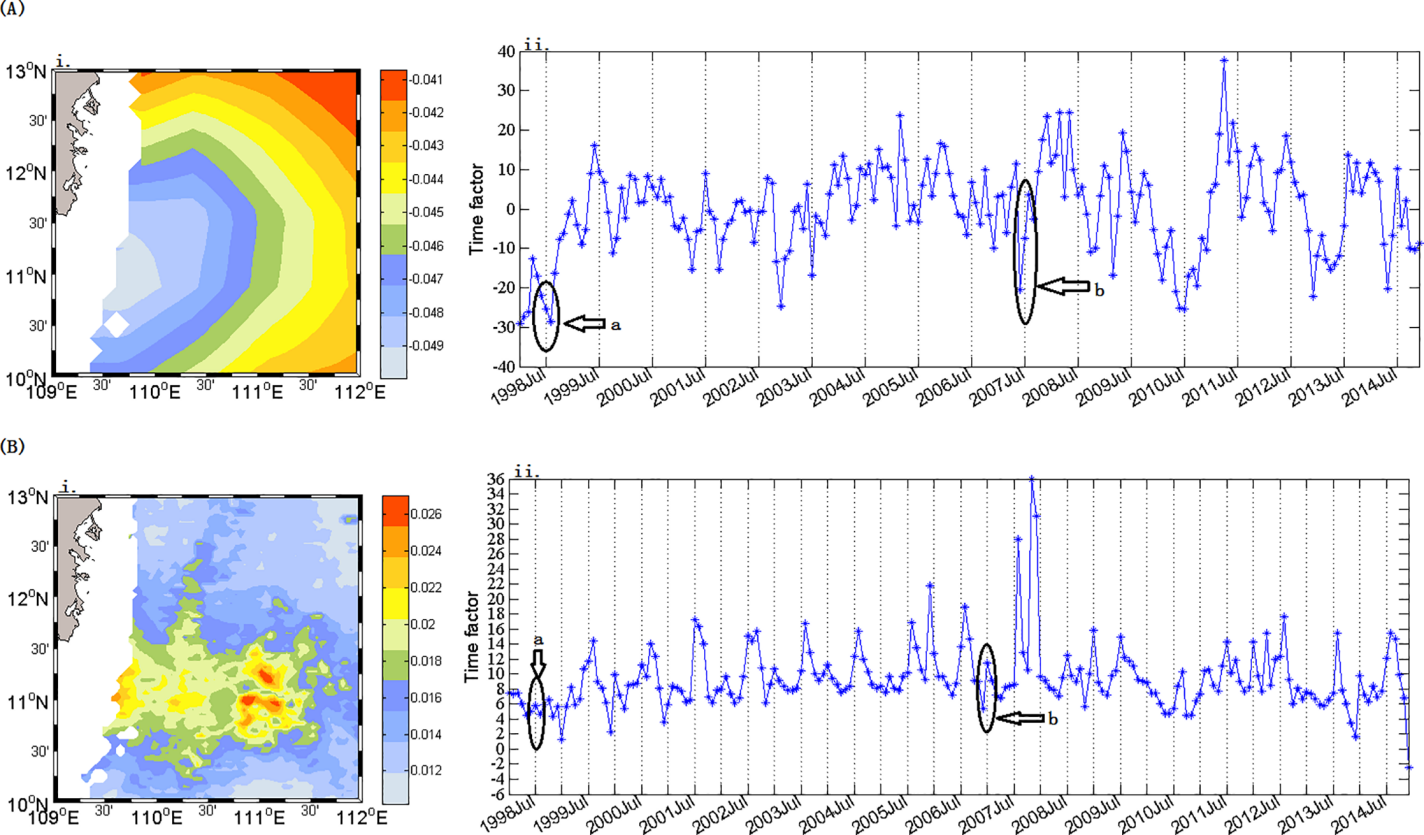
(A) The first EOF modes of SST. A(i) is the spatial field and A(ii) is the time series; (B) The first EOF modes of Chl-a. B(i) is the spatial field and B(ii) is the time series.

The climatology of summer Chl-a concentrations (Fig 2A(iii)) averaged for 1 June—31 August showed substantial spatial heterogeneity in the western SCS. There were generally low Chl-a concentrations in the offshore regions, especially in the offshore deep basin, and high Chl-a in the region southeast of Vietnam, where upwelling is frequent in the summer [4, 29]. The summer Chl-a displayed significant inter-annual variations with different patterns in 1998 and 2007, which were both El Niño years according to the definition of the Climate Prediction Center, NOAA (www.cpc.ncep.noaa.gov/). The first EOF spatial mode of SST (Fig 3A(i)), shows an increase from near shore to offshore. In the first EOF mode of Chl-a (Fig 3B(i)), high concentrations appear in the region of 109.5–111.5°E, 10.5–11.5°N. The first temporal EOF mode of SST (Fig 3A(ii)), accounting for 91.09% of the total variance, showed the highest SST in summers of both 1998 and 2007, however the first temporal EOF mode of Chl-a (Fig 3B(ii)), accounting for 71.01% of the variance, exhibited a different pattern with low values in summer of 1998 and high values in 2007, respectively. The El Niño events in 1998 and 2007 (Table 1) belong to two different types: the former originated in the area of the eastern Pacific Ocean, and the latter in the middle Pacific Ocean, respectively [30].

**Table 1:** El Niño Phenomenon Classification from 1982 to 2016.

Type	Period	Duration (Months)	Max_SSTA* (°C)	Max_Mon**
Eastern type	1982.05–1983.08	16	3.29	1983.01
	1997.05–1998.05	13	3.62	1997.12
	2014.05–2016.04	24	2.93	2015.11
Middle type	1990.11–1992.06	20	0.70	1991.01
	1994.07–1995.04	10	1.01	1994.12
	2002.02–2003.03	14	1.2	2002.11
	2004.07–2005.04	10	0.94	2004.11
	2006.08–2007.02	7	1	2006.11
mixed type	1986.09–1988.02	18	1.76	1987.08
	2009.07–2010.04	10	1.72	2009.12

Max_SSTA*, Max SST Anomaly

Max_Mon**, the month when maximum SSTA occurred.

### Summer Chl-a concentrations during the 2007 El Niño transition year

Low Chl-a in the summer of 2007 (Fig 3A) was generally seen in offshore regions, and southeast of Vietnam showed high Chl-a. However, there was a significantly different pattern during the El Niño year of 1998, with low Chl-a over most of the region. The magnitude of Chl-a in box A in Fig 1 in 2007 was higher than that in 1998–2014, but the area with Chl-a over 0.15 mg m^-3^ was smaller in 2007 than that of 1998–2014. There was a sharp increase in Chl-a concentration from the end of July to mid-August 2007 according to 15-day averaged Chl-a time series of (Fig 2B(v-vi)). From the 15-day spatial distribution of Chl-a in 2007, a band-like area of high Chl-a in early August was also captured by observations.

### Environmental factors

#### Spatial distribution of SST

**Fig 4:**
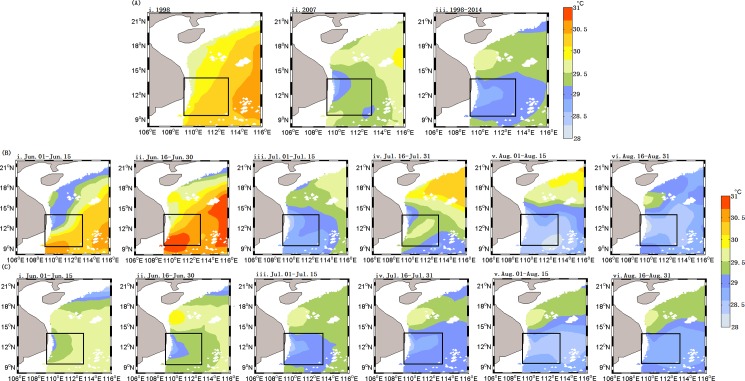
Spatial distribution of SST in the western SCS. (A) Summer SST (averaged for 1 June—31 August), A(i):1998; A(ii): 2007;A(iii): 1998–2014; Time series of SST averaged for 15 days during summer of 2007 (B) and climatology of 1998–2014 (C), respectively.

The summer SST in 1998 (Fig 4A(i)) was higher than that in seen 2007 (Fig 4A(ii)) and higher than the averaged climatology of summer of 1998–2014 (Fig 4A(iii)). The pattern of the summer SST (Fig 4A(i)) was approximately opposite to that of summer Chl-a (Fig 2A(i)) with higher SST at times of lower Chl-a, and vice versa. The increased magnitude of Chl-a was larger in the low SST patch in 2007 than that of the 1998–2014 average. The summer 15-day SST (Fig 4C(i-vi)) averaged for 1998 to 2014 indicated there was a decreasing tendency of SST which reached the minimum in August. However, the summer SST in 2007 (Fig 4B(i-ii)) increased from June to early July with the higher SST than that averaged for 1998–2014, and then declined sharply from July to August with the lowest SST in August (Fig 4B(iii-vi)).

#### Spatial distribution of upwelling

**Fig 5:**
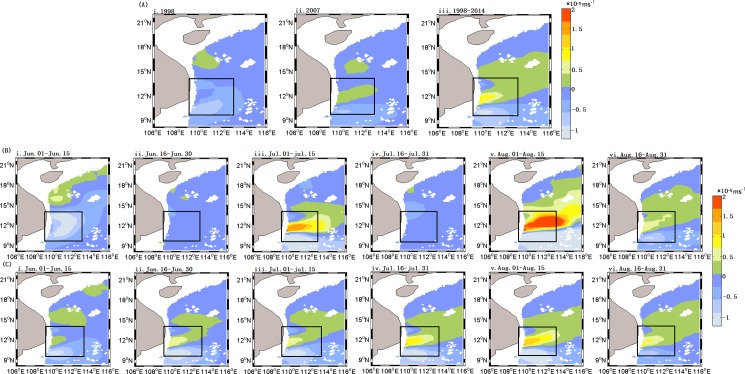
Spatial distribution of Ekman pumping velocity in the western SCS. (A) Summer Ekman pumping velocity (averaged for 1 June—31 August), A(i):1998; A(ii): 2007;A(iii): 1998–2014; (B) and (C) Ekman pumping velocity averaged for 15 days during summer of 2007 and climatology of 1998–2014, respectively.

The summer Ekman pumping velocity intensity averaged for June-August was weaker in 1998 (Fig 5A(i)) than 2007 (Fig 5A(ii)), and both were weaker than the 1998–2014 average (Fig 5A(iii)). However, the 15-day averaged Ekman pumping velocity time series (Fig 5B(i-vi)) indicates that the Ekman pumping velocity in 2007 drastically increased after the end of July and this process was roughly consistent with the increase of Chl-a in 2007 (Fig 2B).

#### Spatial distribution of precipitation

**Fig 6:**
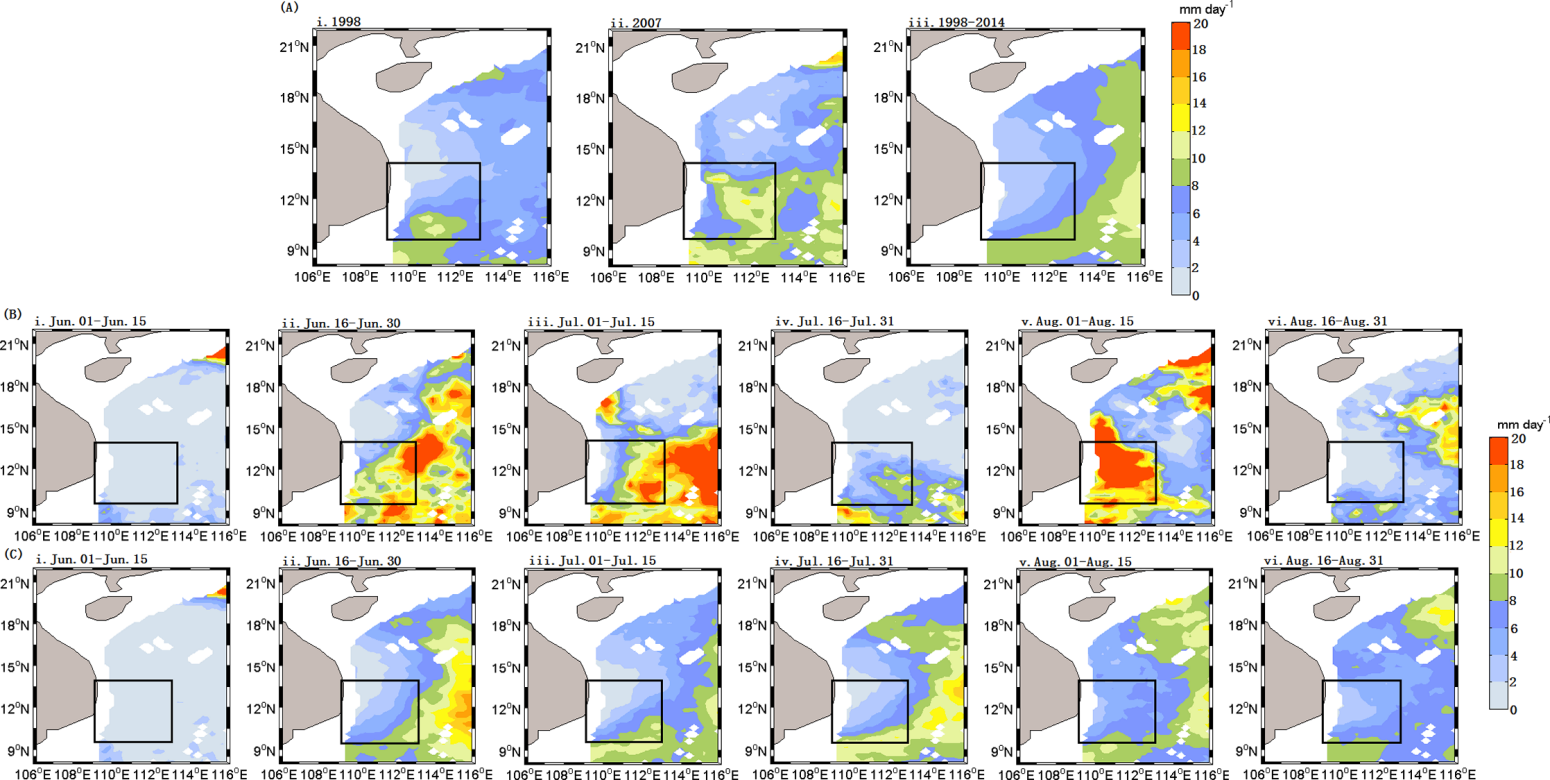
Spatial distribution of precipitation in the western SCS. (A) Summer precipitation (averaged for 1 June—31 August), A(i):1998; A(ii): 2007, A(iii): 1998–2014; (B) and (C) precipitation averaged for fifteen days during the summer of 2007 and the summer climatology of 1998–2014, respectively.

In 2007, the precipitation in the eastern part of Vietnam was stronger, especially in early August, compared with the summer climatology of 1998–2014. The maximum rainfall (Fig 6B(v)) reached 20 mm day^-1^ and the time is roughly the same as the rising time of Chl-a blooms. The average precipitation from 1998 to 2014 is below 8 mm day^-1^ in the east Vietnam, lower than in the open water. The increased precipitation in 2007 may also have provided favorable conditions for the rapid growth of Chl-a.

## Discussion

Nutrients and light are two of the most important factors regulating the biomass and growth of phytoplankton. Considering that the study region is located in the tropical area, nutrients rather than the abundant solar radiation could be the key limiting factor for the growth of the phytoplankton.[4,31]. Complex hydrological/atmospheric conditions in the study area may have considerable influences on the phytoplankton growth, affecting the transportation and distribution of nutrient-rich water in the euphotic layer.

Within the wind driven West African coastal upwelling region from 10°N to 26°N, Roy and Reason [32] demonstrated that the ENSO-induced variability in the Pacific during the early boreal winter could account for a significant part of the variability of coastal SST anomalies measured a few months later. In 2007, the SCS summer monsoon burst during late May in the SCS area was controlled by the southwest wind. The interaction between water vapor flux and the summer monsoon winds in the SCS leads to strong rainfall in North China [33]. Zhou and Chan [34] found that in years associated with a warm (cold) ENSO event, the SCS summer monsoon tends to have a late (an early) onset and the intensity of the SCS summer monsoon also tends to be weaker (stronger) than normal. Low SST, strong upwelling and heavy rainfall from the end of July to early August 2007 occurred in the study area east of Vietnam, which may facilitate uplifting nutrients into the upper layer, triggering the growth of phytoplankton, and high Chl-a concentration [35, 36]. Therefore, these external complex conditions of our study area may be helpful to the transport of nutrients, which may be conducive to the growth of Chl-a.

### Role of Ekman transport and Ekman pumping in summer blooms

**Fig 7:**
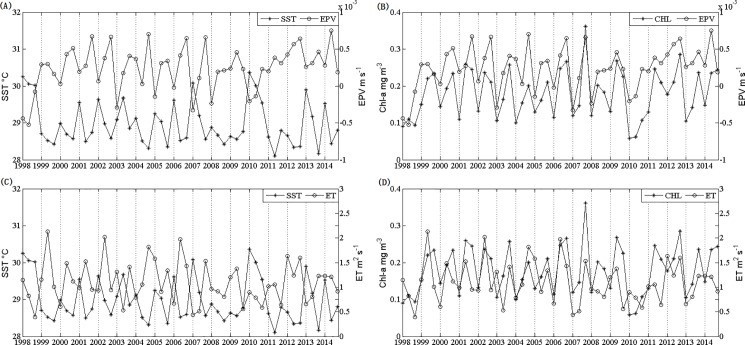
Time series data sample west of the SCS for summer (1 June—31 August) for the period from 1998 to 2014. (A) SST and EPV; (B) Chl-a and EPV; (C) SST and ET; (D) Chl-a and ET.

A correlation analysis of Chl-a and EPV is conducted for the area of 109.3–113°E, 10.8–13.8°N, where high Chl-a concentrations in summer appear frequently. The time series analysis in the ET is implemented over 109–112°E, 9.5–14.5°N, where the strong winds blow generally in the direction parallel to the coastline in summer. In the time series above (Fig 7), there were dramatic changes in Chl-a and other conditions between June and August 2007. In August, the changes in Chl-a and SST were similar to that of the EPV. The correlation coefficient between Chl-a concentration and the Ekman pumping velocity is 0.7338 (p<0.01) (Fig 7B); the correlation coefficient of the Chl-a concentration with the Ekman transport is 0.5118 (p<0.01) (Fig 7D). The multiple correlation coefficient of ET and EPV with Chl-a was 0.7689 (p<0.01), and the partial correlation coefficients were 0.3383 and 0.6680, respectively. These high correlations suggest that both the Ekman pumping velocity and the Ekman transport play important roles in the Chl-a blooms. The influence of the Ekman pumping on Chl-a is more significant than that of the Ekman transport. There are also good correlations of SST with Ekman pumping (r = -0.7095, p<0.01) (Fig 7A), and with the Ekman transport (r = -0.5021, p<0.01) (Fig 7C), implying that the SST is a good index of upwelling, which induces a decrease of SST by pumping low-temperature deep water with high nutrients into the upper layer. Therefore, the above results further indicate that upwelling induced by Ekman pumping and Ekman transport regulated increase of phytoplankton biomass to a large extent. Excluding 2007, the multiple correlation coefficient of SST, ET, and EPV with Chl-a increased to 0.8324 (p>0.01), and the SST partial correlation coefficient was -0.5133, which implies that 2007 was an outlier year with different patterns of wind, temperature and Chl-a.

### Potential factors inducing the summer blooms in 2007

**Fig 8:**
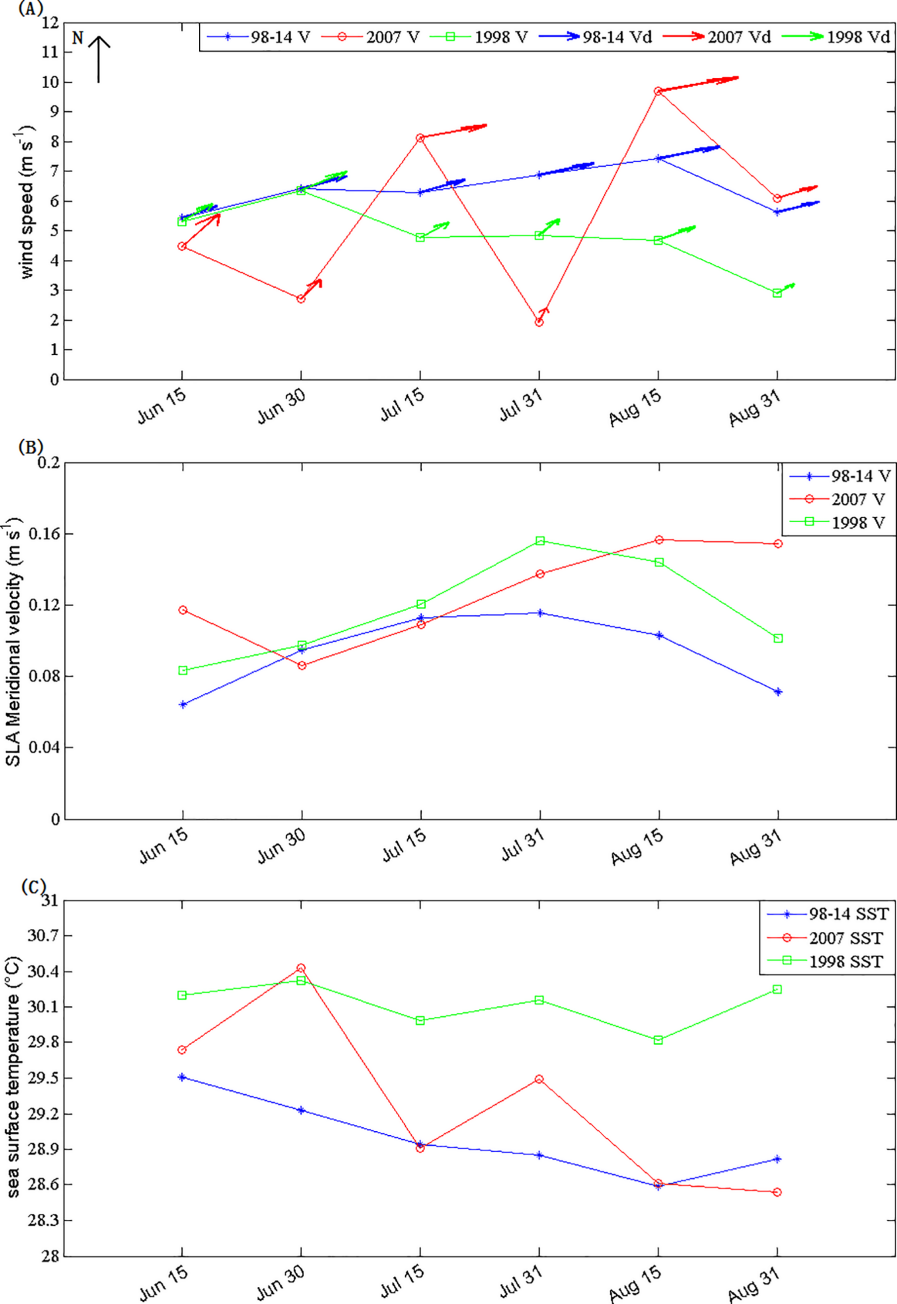
Fifteen-day averaged wind filed (V for speed and Vd for direction), sea level anomaly meridional velocity (V for speed) and SST for Box A during summer in 1998, 2007 and climatology.

The El Niño events of 1997/1998 and 2006/2007 belong to a canonical El Niño and an El Niño Modoki I event, according to the method of Wang and Wang [37], respectively. The trend of SST in 1998 is almost the same as that in climatology, with about 1°C higher than the climatology. However, the change of SST in 2007 was rather strong, with a range of temperature from 30.43°C in late June to 28.54°C in August. SST in June 2007 was higher than that in June 1998, and it is evidently different from that seen during 1998. The 2007 El Nino event was rapidly converted to a La Niña event with sharp decrease of SST (Fig 8C). Similarly, the trend of wind filed in 1998 was roughly consistent with the climatological average. The changes in 2007 are dramatic, and were significantly modulated by the Madden-Julian Oscillation (MJO) (Fig 8A) [38]. The western boundary current (i.e. the Vietnam jet current) [39, 40] (Fig 8B) was strong in summer, and presented evident inter-annual changes in the intensities and the positions of the current, with the jet moving significantly northward. The high Chl-a band in the bloom region had a similar tendency in location and the pattern, indicating the jet may be an important factor in the phytoplankton blooms. In 2007, the meridional velocity derived from sea level anomaly reached its maximum in early August (Fig 8B), which was roughly coincidental with that of the Chl-a bloom. Both the phenomena of the wind-induced summer upwelling [4, 29] and the meridional jet [38, 39] with low SST and high Chl-a have frequently been reported in the study region of the western SCS.

Nutrients are an important factor regulating phytoplankton growth in most regions of the oligotrophic SCS [4, 14]. Under influence of strong solar radiation and weak wind conditions in summer, the stable stratification in the euphotic layer in the SCS inhibits transport of nutrients from the subsurface layer into the near-surface layer. Consequently, a summer subsurface Chl-a maximum (SCM) layer typically appears at a depth between 65 and 85 m, with the euphotic layer reaching a depth of about 120 m [41–43] in the offshore deep water areas of the SCS, due to the absence of nutrients at the near-surface layer and a relatively deep nutricline in the offshore SCS. Therefore, upwelling could not only increase nutrients in the near-surface layer through transporting high nutrients water from the deep layer, but also increase the Chl-a concentrations near the sea surface by shoaling the SCM in a way.

Upwelling may also transport phytoplankton (i.e. Chl-a) from sub-surface to the surface (i.e. shoaling of the SCM layer), resulting in more favorable conditions for phytoplankton growth in the upper layer. The integrated displacement by the enhanced upwelling (Fig 5B(v)) in early August 2007 with the maximum value of 6 m day^-1^ (>5 m day^-1^) is over 40 m, which implies that the simultaneous uplift of nutrients may play an important role in increasing the Chl-a concentration.

## Conclusions

The Chl-a concentration in summer 2007, a transitional period of an El Niño year, was higher than that in a normal El Niño year southeast of Vietnam. The SST declined significantly from June to August 2007, with the steepest decline in early August. The wind speed increased substantially from late July to early August 2007, with the maximum wind parallel to the coast in early August. Dramatically enhanced upwelling was associated with Ekman pumping and offshore Ekman transport due to the significant increase in wind speed might transport high-nutrient and high Chl-a water from subsurface to the sea surface.
